# Grain Refiner Settling and Its Effect on the Melt Quality of Aluminum Casting Alloys

**DOI:** 10.3390/ma15217679

**Published:** 2022-11-01

**Authors:** Gábor Gyarmati, Levente Bogoly, Marcin Stawarz, György Fegyverneki, Zoltán Kéri, Monika Tokár, Tamás Mende

**Affiliations:** 1 Foundry Institute, University of Miskolc, Miskolc-Egyetemváros, 3515 Miskolc, Hungary; 2 Department of Foundry Engineering, Silesian University of Technology, 7 Towarowa Street, 44-100 Gliwice, Poland; 3 Institute of Physical Metallurgy, Metalforming and Nanotechnology, University of Miskolc, Miskolc-Egyetemváros, 3515 Miskolc, Hungary

**Keywords:** aluminum alloy, grain refinement, inclusion, oxide film, bifilm, intermetallic compound, melt treatment, melt quality, sedimentation

## Abstract

Grain refiner particles, which are intended to induce the formation of fine equiaxed grain structure during the solidification of aluminum alloys, are prone to settling during the holding of the liquid metal, which phenomenon can affect not only the grain size but the spatial distribution of the double oxide films in the melt. In this study, the settling of Al_3_Ti inoculant particles, as well as its effects on melt quality and grain refinement, were studied. During the experiments, the Ti-concentration of a liquid Al-Si-Mg-Cu alloy was increased to 0.3 wt.% by the addition of Al-10%Ti master alloy at different melt temperatures. Particle settling and grain size evolution were studied by quantitative metallography, while the interactions of grain refiners and bifilms were investigated by scanning electron microscopy (SEM). The evolution of melt quality was assessed by the computed tomographic (CT) analysis of reduced pressure test (RPT) samples. It was found that effective grain refinement was only realized when the introduced blocky Al_3_Ti particles were dissolved and re-precipitated in the form of (Al,Si)_3_Ti at a lower temperature. Without dissolving at higher holding temperatures, Al_3_Ti particle settling has taken place within 10 min. The settling of (Al,Si)_3_Ti particles improved melt quality by the aided sedimentation of bifilms in the melt.

## 1. Introduction

Adequate control of liquid metal quality and solidification microstructure are essential prerequisites for manufacturing high-quality aluminum castings [1,2,3]. Chemical grain refinement is commonly applied during the processing of aluminum alloy melts to induce the formation of fine equiaxed α-Al grain structure in castings, which usually results in better mechanical properties and reliability [4,5,6,7]. During the addition of grain refiner master alloys, a high number of solid particles are introduced into the liquid alloys that can act as heterogeneous nucleation sites for the primary α-Al dendrites during solidification. In industrial practice, Ti- and B-containing master alloys are used most frequently, which, depending on their chemical composition and manufacturing process, can contain Al_3_Ti, TiB_2_, AlB_12_, and/or AlB_2_ particles [8,9,10,11]. However, after a certain contact time, the grain refining effect of master alloy additions fades, which phenomenon is commonly attributed to the sedimentation, agglomeration, or dissolution of the introduced nucleant particles. Another possibility for reduced refining efficiency is the so-called poisoning effect when an alloying element or impurity reduces the heterogeneous nucleation potency of the introduced particles [12,13,14,15].

Settling of refiner particles in liquid aluminum alloys is one of the most common reasons for decreasing grain refining efficiency with time. The particle sedimentation process is dependent on the density of the solid particles relative to the liquid metal, the size distribution, and shape of the particles, the viscosity of the melt as well as the temperature distribution and convection inside the liquid alloy [16,17,18,19,20]. However, when grain refiner particle settling is considered, it should be taken into account that depending on its processing history, different amounts of native solid phases (inclusions) could be present in the liquid metal that could alter the sedimentation process [21,22,23].

Among the inclusions present in liquid aluminum alloys, double oxide- and nitride films or bifilms should be considered to have a significant effect on particle sedimentation for several reasons. Although it is especially hard to quantify the bifilm population [24,25,26], experimental results [27,28,29] suggest that bifilm number density can be especially high in an average aluminum alloy melt. Campbell [30,31,32,33] estimates a bifilm population of 10^6^ to 10^9^ m^−3^ in an average aluminum melt. If we assume, that each pore is initiated by a bifilm, the authors’ previous results [34] of the computed tomographic (CT) analysis of reduced pressure test (RPT) samples suggest a bifilm population of around 6·10^7^ m^−3^ (60 in 1 cm^3^ of the sample) in a highly contaminated melt. However, this could be an underestimate, as was highlighted by Tiryakioğlu et al. [35], because there could be bifilms that fail to open up and inflate into pores during the solidification of RPT samples. It could be also an overestimate, as more than one pore can be initiated by one bifilm (as in the case of micro-inflated convoluted bifilms [32]). Despite being extremely thin (ranging from a few nm to several μm), the surface area of the oxide layers of bifilms can range from a μm^2^ to several cm^2^ or even dm^2^ [32]. According to Campbell [33], pouring 10 kg of liquid aluminum alloy can introduce 0.1 to 2.0 m^2^ oxide film surface into the melt. Ardekhani and Raiszadeh [36] estimate the average dimensions of bifilm defects in pure liquid aluminum to be 13.8 × 13.8 × 1 μm in the non-convoluted form and between 37 × 37 × 1 μm and 370 × 370 × 0.01 μm if the bifilms are convoluted. Combined with their high number density, bifilms provide surprisingly large areas of solid films that can block the way of settling grain refiner particles. Because of the air gap between their layers, bifilms have nearly neutral buoyancy in liquid aluminum alloys, so grain refining particles contacting them are expected to settle slower [37].

This was experimentally confirmed by Yang et al. [38] who found that by increasing the bifilm quantity by mixing machining chips into liquid aluminum, the sedimentation rate of TiB_2_ particles decreased and the fading time of the grain refining effect increased. In another work, Yang et al. [39] showed that TiB_2_ particles tend to adhere to oxide films, which phenomenon can even prevent TiB_2_ particles from settling. However, maintaining a high bifilm concentration simply to lengthen grain refiner fading would be a faulty and highly uneconomical step. Most (if not all) advantages of grain refinement could be counterweighted by the presence of bifilms [40]: mechanical properties such as ultimate tensile strength, elongation to failure [41,42,43,44,45,46,47] and fatigue limit [48,49,50] could be lowered drastically; fluidity decreases [51,52] while the tendency to porosity and hot tear formation increases by an increment in bifilm content [53,54,55]. This clearly shows that a different approach is needed. Approaching this issue from a different point of view, one may ask, can be the settling of grain refiner particles utilized to facilitate the sedimentation of bifilms suspended in liquid metal? Cao and Campbell [56,57,58,59,60] reported that the settling of primary Fe-rich intermetallic compounds (IMCs) can be utilized to promote the sedimentation of bifilms, as primary α-Al_15_(FeMn)_3_Si_2_ heterogeneously nucleate on the wetted surface of bifilms. The early pioneering work by Mountford and Calvert [61] and the recent studies by Gursoy et al. [62], as well as Gurtaran and Uludağ [63], showed that the settling of Ti-rich compounds can reduce the bifilm content of the upper regions of liquid aluminum alloy melts. Our previous works [64,65] revealed that primary (Al,Si)_3_Ti grain refiner particles are heterogeneously nucleated on oxide films and that during the growth of (Al,Si)_3_Ti particles, oxide film segments became engulfed. In this way, (Al,Si)_3_Ti IMCs were attached to bifilms, which facilitated their mutual settling. However, it is currently not known whether heterogeneous nucleation of grain refiner particles on bifilms and the partial engulfment of oxide films are criteria for bifilm sedimentation or the impingement of particles and bifilms is sufficient to initiate a similar melt-cleaning effect. For this reason, this study aimed to compare the melt cleaning and grain refinement efficiency, as well as the interactions of oxide films and refining particles in two different cases:

1. When the introduced grain refiner particles are dissolved at a higher temperature and then crystallized at a lower temperature, which induces heterogeneous nucleation of particles on the bifilms and the engulfment of oxide film segments.
2. When grain refiner particles are added at a concentration and melt temperature, that does not allow the dissolution of the particles in the liquid alloy.

For this purpose, Al_3_Ti particles were chosen as grain refiner particles, because they can be easily dissolved in liquid aluminum alloys, have a tendency to settle in the melt, and are widely used as grain refiner particles or as a reinforcing phase in aluminum matrix composites [66,67,68]. In this way, the results of the present investigation could be useful not only in terms of bifilm sedimentation but regarding the application of grain refiners and the manufacturing of Al_3_Ti-reinforced aluminum matrix composites.

## 2. Materials and Methods

**Figure 1:**
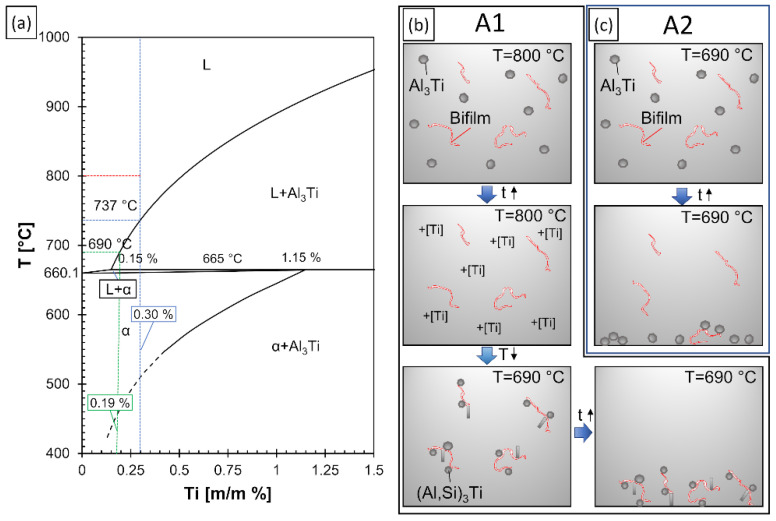
(**a**) The aluminum-rich section of the Al–Ti binary phase diagram (adapted from [69]), (**b**,**c**) schematic illustration of particle and bifilm settling during the experiments.

Two experiments were conducted, which are labeled A1 and A2. In both cases, 3 kg of EN AC-45500 alloy (with an initial composition of 7.1% Si, 0.49% Cu, 0.38% Mg, 0.13% Fe, 0.12% Ti; all compositions are given in weight percentage until stated otherwise) was melted in a resistance-heated crucible furnace (equipped with an A10 clay-graphite crucible) at 690 ± 5 °C. To increase the oxidation rate of the alloy (which can make it easier to detect bifilms due to their faster thickening), the Mg concentration was raised to 0.7% by alloying with commercial purity Mg. For melt quality evaluation, reduced pressure test (RPT) samples were cast at 80 mbar pressure. The sampling of the specimens was realized by the immersion of steel cups into the liquid metal to minimize bifilm formation. Following the casting of 2 control RPT samples, two different approaches were used for the introduction of Al_3_Ti particles (Figure 1b,c). 

For experiment A1, the melt temperature was first raised to 800 ± 5 °C, which temperature was reached under 10 min. To reach 0.3% Ti-concentration, Al-10%Ti master alloy was added, followed by a 1-min-long stirring for the homogenization of the alloy composition. After a 10-min-long holding, the temperature was lowered to 690 ± 5 °C to initiate the crystallization of (Al,Si)_3_Ti particles (Figure 1b). This temperature was reached under 14 min.

In the case of the A2 experiment, the same amount of Al-10%Ti master alloy was added to the melt, but at a constant 690 °C holding temperature to minimalize the dissolution of Al_3_Ti particles. Based on the binary Al-Ti phase diagram (Figure 1a), at 690 °C the liquid phase can dissolve 0.19% Ti, and 0.15% Ti can remain in solution when the temperature is lowered to the peritectic temperature. The initial Ti concentration was 0.12%, so a maximum of 0.07% Ti could be dissolved from the master alloy at 690 °C. It should be noted that the presented solubility values are approximated as the alloying elements Si and Mg in Al-Si alloy will affect the solubility of Ti in molten Al-Si alloy. After the addition of the master alloy, 1-minute long mixing and 10 min long holding was applied. In the case of the A1 experiment, Figure 1a predicts the formation of primary Al_3_Ti, when the melt temperature is lowered from 800 °C to 737 °C. However, as the alloy used is not binary Al-Ti, the formation temperature may differ. Chen and Fortier [70] used melt quenching and LiMCA (Liquid Metal Cleanliness Analyser) methods to study the crystallization temperature of (Al,Si)_3_Ti particles for multiple alloys with different Ti concentrations. For the Al-7%Si-0.35%Mg (A356) alloy with 0.26% Ti, the melt quenching experiments gave a formation temperature of 724 °C, while the LiMCA method predicted 750 °C. Regardless of the possible inaccuracies, it is evident that by lowering the melt temperature from 800 °C to 690 °C, the crystallization of primary (Al,Si)_3_Ti was achieved.

During both experiments, 5 RPT specimens were cast after Ti-alloying at approximately 7-min-long time intervals. In the case of A1, the sampling started immediately after the temperature was lowered to 690 ± 5 °C, while during A2, the sampling started after the 10-minutes-long holding period. Samples were cast for optical emission spectroscopy (OES) before and after Mg-alloying, as well as after the Ti-alloying step and the 10-minutes long holding. ARL iSpark 8820 OES (Thermo Scientific™, Dreieich, Germany) apparatus was used for the OES analysis. After sampling, the remaining melt solidified in the furnace with a 0.9 °C⋅min^−1^ cooling rate. After standard metallographic preparation, the microstructural features were studied with optical microscopy (OM) (Zeiss Axio Observer 3, Carl Zeiss Microscopy GmbH, Jena, Germany) and scanning electron microscopy (SEM). The SEM investigations were realized with a Zeiss EVO MA 10 scanning electron microscope (Carl Zeiss Microscopy GmbH, Jena, Germany) equipped with an EDAX energy-dispersive X-ray spectroscopic (EDS) detector. Image analysis of optical micrographs was used for the characterization of the grain refiner particles in the Al-10%Ti master alloy and the sediment layers formed during the experiments. For this purpose, ImageJ software was used. The average height of the particle-rich sediment layer was evaluated by dividing the microstructural images of the bottom region of the samples into 500 μm wide columns and measuring the distance between the bottom of the sample and the grain refiner particles at the highest point of each column. The average result of 50 measurements was used for the characterization of the sediment layer. Cross-sectional chemical analyses were made with a Horiba Jobin GD-Profiler 2™ (Horiba France SAS, Longjumeau, France) glow discharge optical emission spectrometry (GDOES) apparatus. 

The CT analysis of the RPT samples was executed with a GE Seifert X-Cube Compact 225 kV ( GE Sensing & Inspection Technologies GmbH, Ahrensburg, Germany) apparatus (135 kV, 0.8 mA) and VGSTUDIO MAX 3.2 software. The VGDefX algorithm was used for the detection of the pores. A probability value was determined for each pore, which depends on the local grey level contrast. During the analysis, pores with a volume smaller than 0.05 mm^3^ and with a probability value lower than 0.5 were ignored. As the pore number density, size and distribution can be affected by the grain refinement effect of the (Al,Si)_3_Ti particles [71,72,73], the average grain size of the α-Al dendrites in the RPT specimens was also investigated. Metallographic sections were prepared at 1 cm height of the RPT samples, then were anodized with Barker’s reagent (Struers LectroPol-5 apparatus (Struers LLC, Detroit, MI, USA), 5 g HBF4 + 200 mL distilled water, 25 V dc, and 40 s etching time). The linear intercept method was used for the calculation of mean intercept length, and the grain number density was also calculated according to the ASTM E112-12 standard [74]. For the calculation of grain number density, the number of grains was divided by the area of α-Al dendrites, which was determined by ImageJ software. The sample taken before Ti-alloying, as well as the 1st, 3rd, and 5th, specimens cast after Ti-alloying were investigated this way. Throughout the analysis of the results of the present study, the time starting from the beginning of the experiment will be labeled as time_1_, while time_2_ represents the time starting from the moment when the melt temperature decreased to 690 °C after Ti-alloying (A1) or the moment when the holding period after alloying has ended (A2).

## 3. Results and Discussion

### 3.1. Grain Refinement

**Figure 2:**
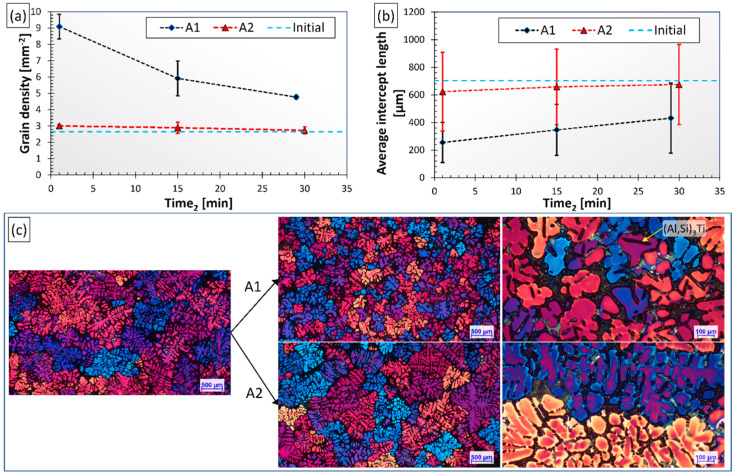
(**a**) Average grain number density and (**b**) mean intercept length results, (**c**) grain structure of the RPT sample cast before and after Ti-alloying for both experiments.

The results of the grain size analysis are summarized in Figure 2. Figure 2a,b present the evolution of average grain number density and average intercept length results, while Figure 2c shows microstructural images of the samples cast before and after Al-10%Ti addition for both experiments. The initial grain density and intercept length values measured on the control samples are represented by blue dashed lines for easier comparison. 

There is an evident difference between the degree of grain refinement achieved in the course of the two experiments. During the A1 experiment, the dissolution of the introduced Al_3_Ti particles at 800 °C and the precipitation of (Al,Si)_3_Ti particles at 690 °C resulted in significant grain refinement: the average intercept length reduced from about 700 μm to 250 μm with a grain density increment from 2.62 mm^−2^ to 9.09 mm^−2^. This is the result of numerous small-sized (Al,Si)_3_Ti particles with flake-like morphology, which could be found inside the α-Al dendrites (see Figure 2c) indicating that the particles acted as nucleation sites. With time, the grain refinement effect was gradually reduced, presumably due to the settling of (Al,Si)_3_Ti particles. In the case of experiment A2, no significant grain refinement was achieved even though the same amount of grain refiner was added as during the A1 experiment. In this case, there were no intermetallic particles found inside the dendrites.

The grain refining effect of Al-Ti master alloys was studied in several different studies. Samuel et al. [75] studied the grain refining effect of different grain refiner master alloys in the case of an A356.2 alloy. When Al-10%Ti master alloy was used, several different Ti concentrations, ranging from 0.02% to 0.5%, were tested. The Ti-alloying was made at 750 ± 5 °C and by increasing the Ti-concentration, the grain size was reduced from about 450 μm to 300 μm. Similarly to the case of our A1 experiment, flake-like (Al,Si)_3_Ti particles were found in each sample; only the size and number density were different at different Ti levels. In another work, Samuel et al. [76] studied the grain refining effect of Al-10%Ti master alloy (containing flake-like Al_3_Ti particles) in the case of A356.2 alloy, with Ti additions ranging from 0.1% to 0.6% at holding temperatures of 750 °C and 950 °C. Regardless of the melt temperature, the addition of 0.15–0.2% Ti resulted in the reduction of grain size from about 1850 μm to 600 μm. Higher Ti-concentrations also resulted in an average grain size of about 600 μm. Like in our case, (Al,Si)_3_Ti particles were found inside the α-Al dendrites suggesting that the particles acted as nucleation sites. During a similar experiment, with the same experimental conditions and alloy, Samuel et al. [77] found that the alloy grain size decreases linearly (from 3700 μm to 710 μm) with the increase in the Ti concentration up to about 0.2%Ti followed by a steady state stage, with no further increase in grain refining regardless of melt temperature (750 °C, 950 °C or 950 °C for 30 min followed by casting at 750 °C). Tahiri et al. [78] used the same sample geometry, melt temperature, and base alloy as Samuel et al. [77] to study the grain refinement effect of different master alloys, including Al-10%Ti with Ti concentrations ranging from 0.1% to 0.4%. The best grain refining effect was achieved with the utilization of 0.2% Ti, which reduced the initial 1855 μm grain size to about 400 μm. In the case of 0.3% Ti level, the grain size was 650 μm, which is much higher than the 250 μm achieved during our A1 experiment. However, it should be mentioned that the cooling rate of the specimens and the exact chemical composition were different from in our case. Some of the above-mentioned studies reported that the melt temperature had no significant influence on the effectiveness of the grain refinement with Al-Ti master alloys, which is contradicting the results of the present work. This is most probably due to the differences in the experimental conditions of the mentioned studies and the present work: Samuel et al. [75,76,77] and Tahiri et al. [78] used continuous melt stirring to avoid particle sedimentation, and it is also possible that the microstructure (including morphology and degree of agglomeration of Al_3_Ti particles) of the used master alloys was different.

### 3.2. Grain Refiner Particle Settling

The most reasonable explanation for the inefficient grain refinement during experiment A2 is that the introduced Al_3_Ti particles settled before the first RPT sample was taken. This is supported by the results of the OES analysis of the samples taken at different stages of the experiments (Table 1).

**Figure 3:**
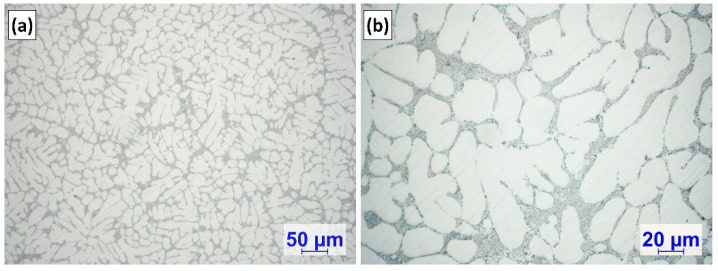
(**a**,**b**) Microstructural images of the OES sample cast 10 min after Ti-alloying during the A2 experiment.

Table 1 presents 3 rows of chemical composition values for both experiments: the first (A1/1 and A2/1) was measured after melting, the second (A1/2 and A2/2) after Mg-alloying, while the third (A1/3 and A2/3) 10 min after Ti-alloying. As can be seen, there is an outstanding difference between the Ti-concentrations of the Ti-alloyed samples (A1/3 and A2/3). During the A2 experiment, instead of the desired 0.3% Ti-content, only 0.147% was reached, which indicates that there were no Al_3_Ti particles in the upper region of the melt, which was used for casting the 3rd OES sample 10 min after Ti-addition. This is confirmed by the optical microscopic investigation of the OES sample (Figure 3).

**Figure 4:**
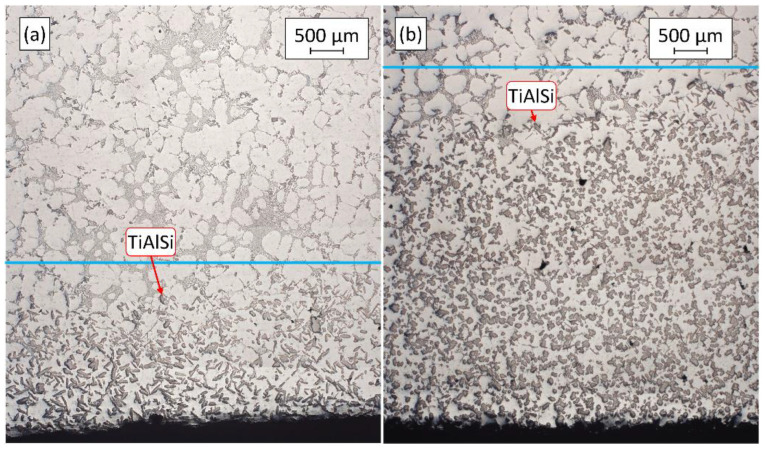
Settled TiAlSi particle-rich zones formed during (**a**) experiment A1 and (**b**) A2.

Figure 4 shows the optical micrographs of the settled TiAlSi particle-rich bottom section of the samples solidified in the crucibles. In the figure, blue lines are used for the visualization of the maximum height where grain refiner particles are found. Table 2 presents the results of the analysis of the height of particle-rich zones.

**Figure 5:**
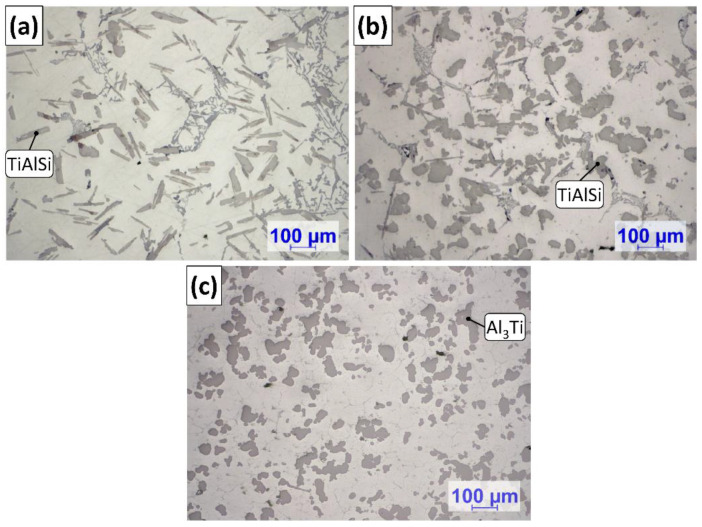
Optical micrographs of the TiAlSi particles formed during the (**a**) A1 and (**b**) A2 experiments, as well as the (**c**) microstructure of the Al-10%Ti master alloy.

**Figure A1:**
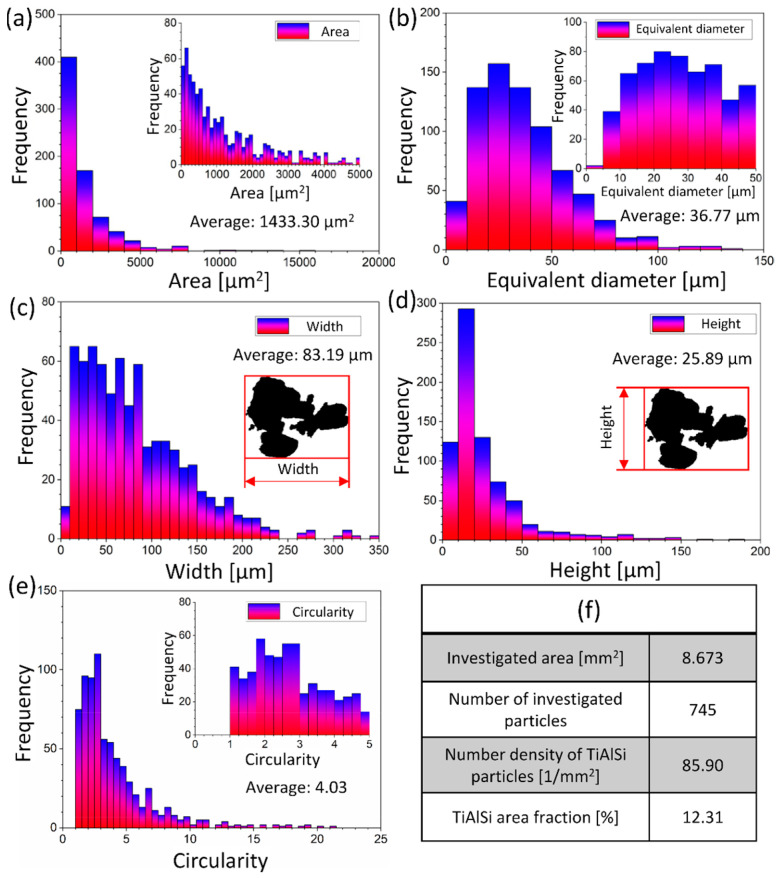
(**a**–**f**) Results of the image analysis of TiAlSi particles in the A1 sample.

**Figure A2:**
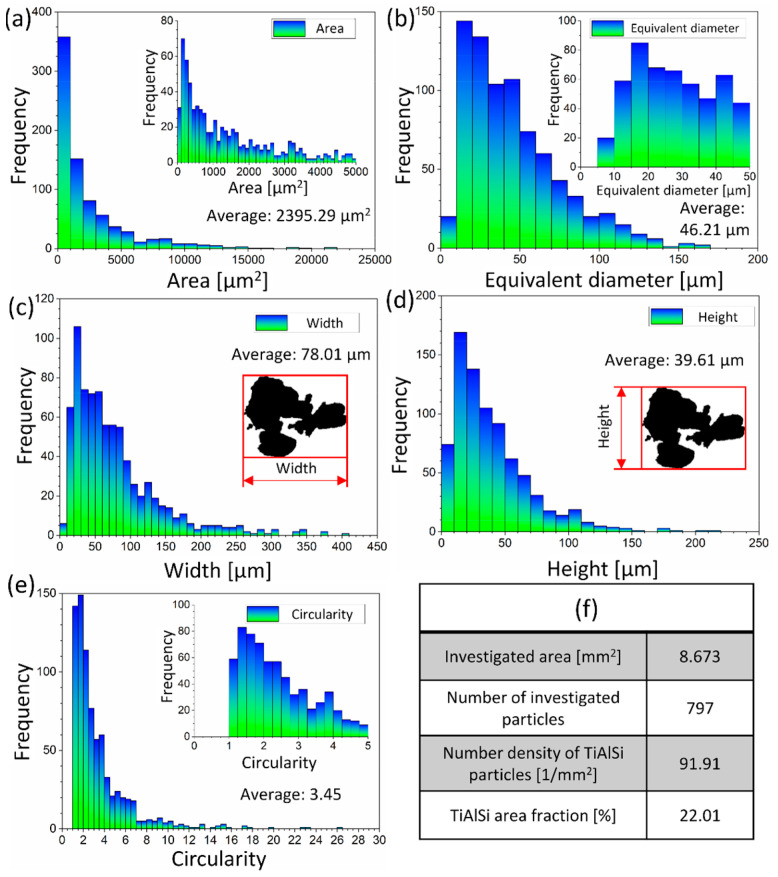
(**a**–**f**) Results of the image analysis of TiAlSi particles in the A2 sample.

**Figure A3:**
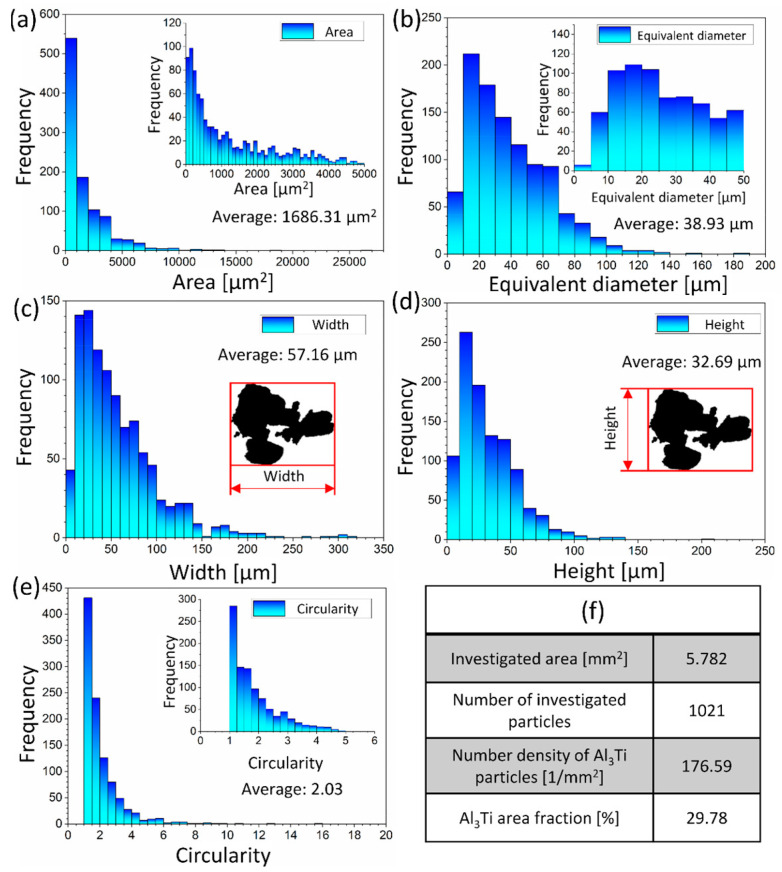
(**a**–**f**) Results of the image analysis of Al3Ti particles in the Al-10%Ti master alloy.

It can be clearly seen in Figure 4 and the results in Table 2 that during the A2 experiment the thickness of the sediment layer was remarkably larger. This suggests that more particles reached the bottom region of the melt during the A2 experiment, than in the case of A1. Besides the height of the sediment layer, there were significant differences in the morphology of the TiAlSi particles. Figure 5 shows optical micrographs of the settled grain refiner particles for the A1 (Figure 5a) and A2 (Figure 5b) experiments. The microstructure of the Al-10%Ti master alloy is also presented for comparison (Figure 5c). The detailed results of the quantitative image analysis of the particles are given in Appendix A (see Figure A1 for experiment A1, Figure A2 for experiment A2 and Figure A3 for the Al-10%Ti master alloy).

The Al_3_Ti particles in the Al-10%Ti master alloy have blocky morphology, which is a consequence of the production parameters of the master alloy [79]. In contrast, the TiAlSi particles formed during the A1 experiment have a plate-like morphology, which indicates that during the holding period at 800 °C, the blocky Al_3_Ti particles dissolved, and by lowering the temperature to 690 °C, flake-like (Al,Si)_3_Ti particles were crystallized. The Al_3_Ti particles added during the A2 experiment mostly retained their blocky morphology (Figure 5b), because they did not dissolve during the experiment. However, some parts of the particles became lamellar, which is a sign of a reaction that involves morphological transition.

**Figure 6:**
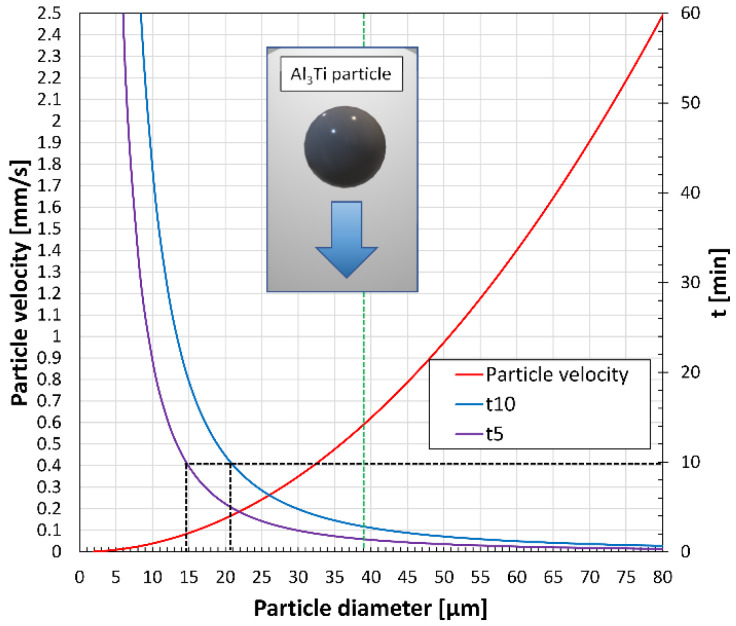
Terminal particle settling velocity values and the time needed to settle 5 and 10 cm as a function of the diameter of spherical Al_3_Ti particles.

As it was mentioned above, the degree of dissolution of the Al_3_Ti particles introduced into the melt during the A2 experiment was negligible and particle settling was remarkable after 10 min of holding time. If we assume that no initial force was acting on the particles except for gravity; the particles are spherical and not agglomerated, and the only drag force considered is the force between the liquid metal and the particles; the wall effect and convection effect can be ignored; then the settling velocity of the particles can be estimated by using Stokes’ law [16]:

(1) $$v=\frac{2r^{2}\left(\rho_{p}-\rho_{l}\right)g}{9\mu }$$

where v is the terminal velocity of the settling particle [cm/s], r is the radius of the spherical particle [cm], $\rho_{p}$ is the density of particles (3.130 g/cm^3^ for Al_3_Ti [16]), $\rho_{l}$ is the density of the liquid alloy at 690 °C (2.385 g/cm^3^ [80]), g is the acceleration due to gravity (981.7 m/s^2^), and μ is the viscosity of the liquid alloy at 690 °C (0.0104 g/cm·s [80]). Figure 6 presents the calculated settling velocities as a function of particle diameter indicating the average equivalent diameter of the Al_3_Ti particles inside the Al-10%Ti master alloy (~39 μm). Based on the terminal velocity values, the time needed to settle 5 and 10 cm (t5 and t10) was also calculated. The reason for this is that during the experiments, the total height of the melt was around 10 cm after Ti addition. However, a settling distance of a few centimeters could also result in the low Ti concentration measured during the A2 experiment (Table 1), as the OES sample was taken from about 3–4 cm below the melt surface. 

Based on Figure 6, the settling rate of the particles is increasing with particle diameter, which results in lower t10 and t5 values. At the average equivalent diameter of 39 μm, v = 0.59 mm/s, t10 = 2.82 min, and t5 = 1.4 min, which means that particles with at least 39 μm diameter will settle to the bottom of the melt within less than 3 min. During the 10 minutes-long holding period after alloying, particles with at least 21 μm diameter will settle fully, while a diameter of 15 μm will result in a 5 cm settling distance. Based on the particle size distribution (Figure A3) about 83% of the Al_3_Ti particles have a larger equivalent diameter than 15 μm, which suggests that the degree of sedimentation could be extensive. The sedimentation process could be even faster if the agglomeration of the particles is also considered. For example, Schaffer and Dahle [81] found that TiB_2_ grain refiner particles tend to settle much faster than predicted by Stokes’ law due to the agglomeration of the particles. Based on these, it is highly possible that particle settling was significant 10 min after the introduction of the master alloy during experiment A2.

### 3.3. Phase Evolution of Grain Refiner Particles

**Figure 7:**
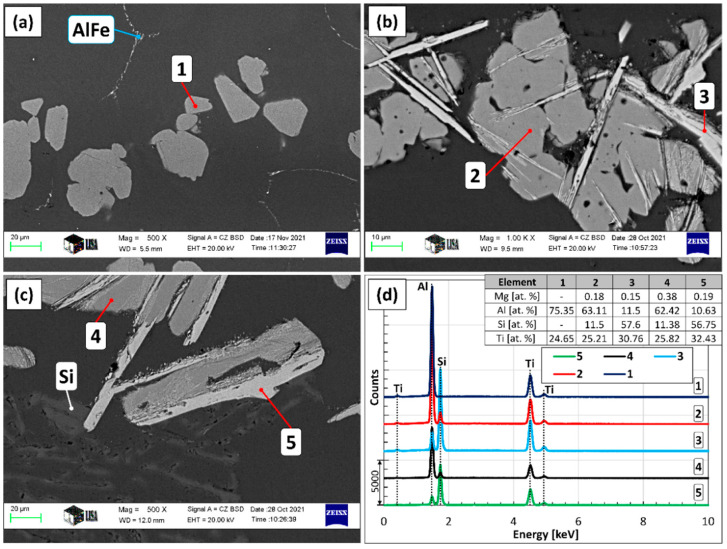
(**a**) SEM image of the Al-10%Ti master alloy, (**b**) the particles formed during experiment A2 and (**c**) A1, (**d**) the results of the EDS analysis at the indicated points.

Figure 7 shows the SEM images of the grain refiner particles found in the Al-10%Ti master alloy (Figure 7a), as well as in the samples solidified in the crucible during A1 (Figure 7c) and A2 experiments (Figure 7b). The results of the EDS analyses are given in Figure 7d. Based on the SEM investigation and the EDS analyses, the chemical composition of the grain refiner particles in the master alloy is homogeneous and follows the Al_3_Ti stochiometry (75 at.% Al and 25 at.% Ti, point 1 in Figure 7). On the contrary, the particles created during the two experiments consist of two phases: an (Al,Si)_3_Ti phase that is lower in Si and Ti (points 2 and 4 in Figure 7), as well as a phase that has a relatively high Si-concentration (around 57 at.%, point 3 and 5 in Figure 7). Based on the results of our previous studies [64,65] and references [82,83,84], the latter is the τ_2_ phase, which is created by the transformation of the (Al,Si)_3_Ti during the slow cooling of the samples.

**Figure 8:**
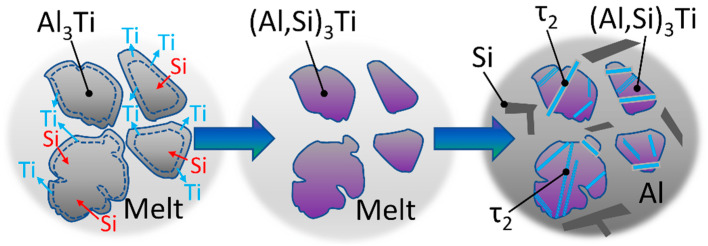
Transformation of Al_3_Ti particles during the A2 experiment.

It can be seen in Figure 7b, that the parts of the particles that transformed to τ_2_ have a lamellar morphology. Based on this, the particles created during the A2 experiment differ from their original blocky morphology due to the phase transition to τ_2_. Ma et al. [83] reported that under equilibrium conditions, τ_2_ can appear as a result of the $L+{\left(\mathrm{Al},\mathrm{Si}\right)}_{3}\mathrm{Ti}\to \mathrm{Al}+\tau_{2}$ reaction in the case of alloys with similar Si concentrations as in our case. This is realized via the diffusion of Si atoms into the (Al,Si)_3_Ti particles, as well as the local enrichment of Si and Ti atoms inside the particulates. This results in the formation of a lamellar structure inside the blocky (Al,Si)_3_Ti particles. This reaction is commonly suppressed during the solidification of cast alloys and composites due to non-equilibrium cooling conditions. The transformation can be also realized via solid-state transformation, which is a slower process and can be neglected in our case. The transformation sequence of the Al_3_Ti particles introduced to the liquid alloy during the A2 experiment is summarized in Figure 8.

First, the partial solution of the introduced Al_3_Ti particles raised the Ti-concentration of the liquid metal, until the solubility limit at 690 °C was reached. The solute Si diffused into the Al_3_Ti particles, transforming them to (Al,Si)_3_Ti particulates. It is reported that up to 15 at.% Si can be dissolved in the crystal lattice of Al_3_Ti by the substitution of Al atoms, forming (Al,Si)_3_Ti. First, a (Al,Si)_3_Ti shell is formed on the surface of the Al_3_Ti particles, which is gradually transformed into (Al,Si)_3_Ti by the inward diffusion of Si [82,83]. In the course of the A2 experiments, most of the introduced grain refiner particles had settled. During the solidification of the sample, the phase transition into τ_2_ has taken place after the α-Al dendrites formed. This resulted in the formation of the lamellar τ_2_ phase. As the transformation is controlled by the diffusion of Si atoms into the particles from the liquid metal, as well as the diffusion of Si and Ti atoms inside the particles, the transformation is a time-consuming process that had been not fully realized during the experiment.

### 3.4. Interactions of Oxide Films and Grain Refiner Particles

**Figure 9:**
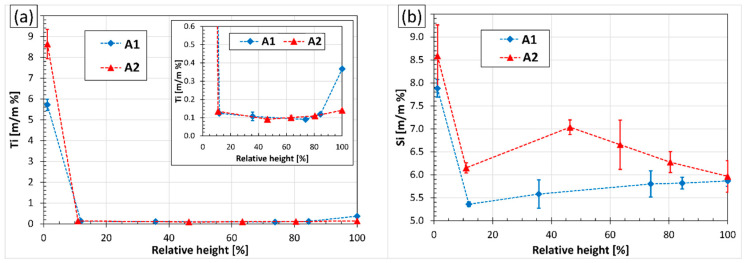
(**a**) Ti- and (**b**) Si-concentrations as a function of relative height in the samples solidified in the crucible during the experiments.

Figure 9 shows the results of the GDOES analyses made on the cross-section of the samples solidified in the crucibles during the two experiments. Figure 9a presents the concentration distribution of Ti, while Figure 9b shows the Si concentrations measured at different heights. The heights of the samples were 84 mm and 82 mm for A1 and A2, respectively. At the bottom region of the samples, both Ti and Si were enriched because of the high number of TiAlSi grain refiner particles settled during the experiments. In the case of the A2 experiment, the Ti- and Si-concentrations were higher, which is caused by the higher number density of TiAlSi particles than in the case of the A1 experiment. In both samples, above the Ti- and Si-rich zone, the Si-concentration dropped significantly, which can be explained by the (Al,Si)_3_Ti→τ_2_ phase transformation, which involves the diffusion of Si from the liquid metal to the (Al,Si)_3_Ti particles.

**Figure 10:**
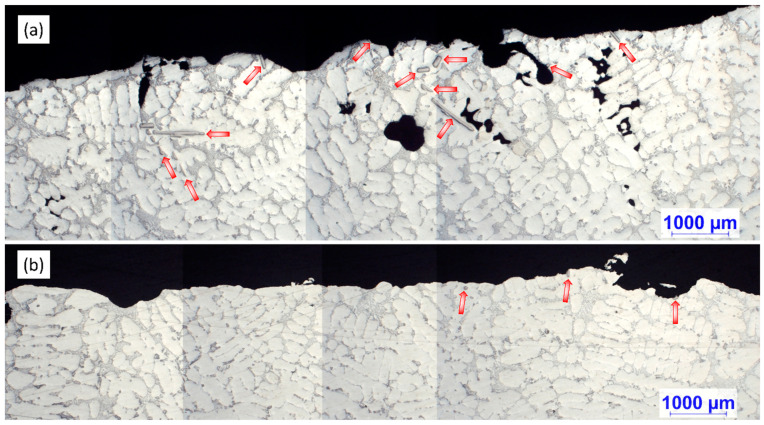
Optical micrograph of the top region of the (**a**) A1 and (**b**) A2 samples, indicating the places where TiAlSi particles were found.

There is an important difference between the composition at the top region of the sample: in the case of A1, Ti is enriched near the top surface of the specimen to a degree (0.37%) that indicates the presence of TiAlSi compound particles. Indeed, the microscopic investigation of this region (Figure 10a) revealed the presence of numerous TiAlSi particles that seem to be attached to the surface oxide layer and its entrained parts. As it was detailed in our previous study [64], this attachment is caused by the heterogeneous nucleation of (Al,Si)_3_Ti particles on the surface oxide film and the partial engulfment of some parts of the oxide films during the growth of the particles.

In the case of A2, there is no significant Ti-enrichment at the top region of the sample (the Ti-concentration is 0.14%) and the microscopic analysis (Figure 10b) revealed a negligible amount of extremely small TiAlSi particles. These were presumably created when the sample was cooling from 690 °C (after all RPT specimens were taken) and the Ti-solubility of the liquid alloy was decreased. Based on this, the Al_3_Ti particles introduced during alloying were not attached to the surface oxide layer.

**Figure 11:**
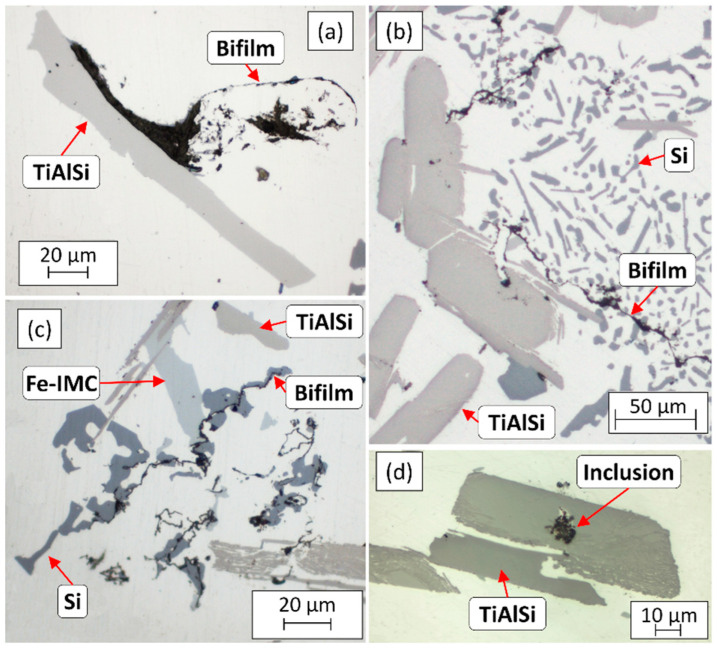
(**a**–**d**) Optical micrographs of inhomogeneities found in the sediment formed during the A1 experiment.

**Figure 12:**
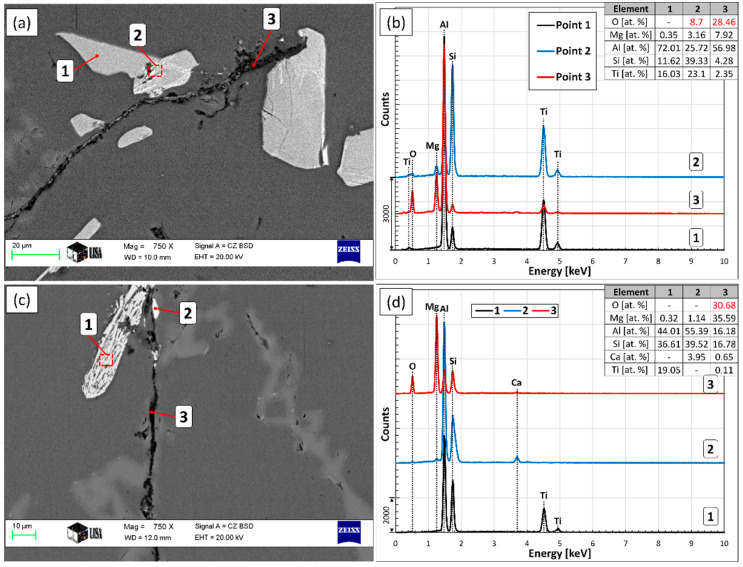
(**a**,**c**) SEM images of bifilms (point 3 in both images) and intermetallic particles (points 1 and 2), (**b**,**d**) results of the EDS analysis at the indicated points.

During the microscopic examination of the bottom region of the A1 sample, numerous crack-like bifilms were found, which were connected to Si, Fe-rich intermetallic, and TiAlSi compound particles (Figure 11). In one case (Figure 11d), an inclusion was also found inside a TiAlSi particle, which appears to be an agglomerate of inclusion particles; however, it could also be a bifilm that was raveled up into a compact form. Figure 12 presents the SEM images of two bifilm defects found in the sediment zone (Figure 12a,c) and the results of the EDS analysis made at the indicated points (Figure 12b,d).

In Figure 12a, particles consisting of (Al,Si)_3_Ti (point 1) and τ_2_ (brighter regions inside the particles, point 2) are attached to the oxygen-rich crack-like feature (point 3). Based on its O- and Mg-concentration, the investigated inhomogeneity is a MgAl_2_O_4_ film, which is thermodynamically more stable than MgO or Al_2_O_3_ for the studied alloy composition and temperature [85]. Note that during the analysis of point 2, some parts of the darker regions inside the particle were also inside the electron-excited interaction volume, which resulted in the detection of some oxygen. Based on this, there is an oxygen-containing phase inside the investigated particle. The white particles inside the bifilm are presumably fragments of the TiAlSi compounds formed during the sample preparation. 

In Figure 12c, a TiAlSi particle (point 1) and a Ca-containing intermetallic particle (point 2) are connected to the studied inhomogeneity (point 3). The TiAlSi particle is apparently fully transformed to τ_2_ lamellae and Al phase according to the $L+{\left(\mathrm{Al},\mathrm{Si}\right)}_{3}\mathrm{Ti}\to \mathrm{Al}+\tau_{2}$ reaction. The O- and Mg concentration of the crack-like feature is relatively high and indicates the presence of MgO. This is probably the result of the Mg-alloying process in which, pure Mg was plunged into the liquid alloy. The presence of the Ca-containing intermetallic compound particle can be explained by the interfacial segregation of the surface-active Ca and the heterogeneous nucleation of the compound particle on the oxide film. A similar phenomenon was reported by Al-Helal et al. [86] for Al_2_CaSi_2_, as well as by Chen and Griffith [87] for Al_2_SrSi_2_ compound particles.

**Figure 13:**
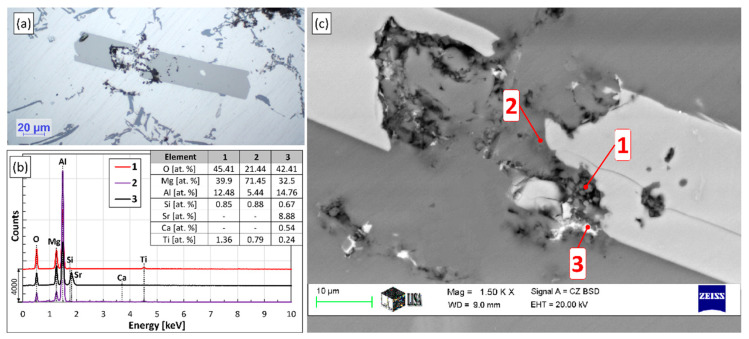
(**a**) Optical micrograph and (**c**) SEM image of an (Al,Si)_3_Ti particle associated with oxide phases, (**b**) the results of the EDS analysis at different points.

In the case of the (Al,Si)_3_Ti particle presented in Figure 13, oxide phases were found not only attached to the particle but also inside it (darker regions in Figure 13a,c). This is caused by the engulfment of the oxide phase during the growth of the particle, and it is in accordance with our previous study [64] where this phenomenon was also reported. 

The investigated O-rich phases had rather different chemical compositions (Figure 13b): in points 1 and 2, the main constituents are O and Mg, but their ratio is different. In point 3, besides Mg and O, Sr and Ca were also found, which can be explained by the segregation and enrichment of surface-active Ca and Sr along the oxide phase.

**Figure 14:**
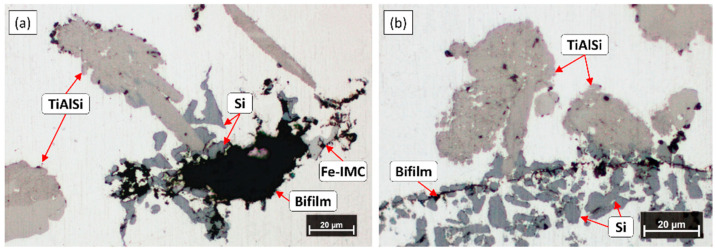
(**a**,**b**) Optical micrographs of bifilms found in the sedimented particle-rich regions formed during the A2 experiment.

Bifilms were also found during the investigation of the sediment layer formed during the A2 experiment. In these cases, TiAlSi intermetallics were rarely connected to the bifilms, instead, Si and Fe-rich intermetallic particles were found to become attached to the oxide films (Figure 14), which suggests that these phases nucleated on bifilms and TiAlSi particles only collided with them during the sedimentation process.

### 3.5. Evolution of Melt Quality

**Figure 15:**
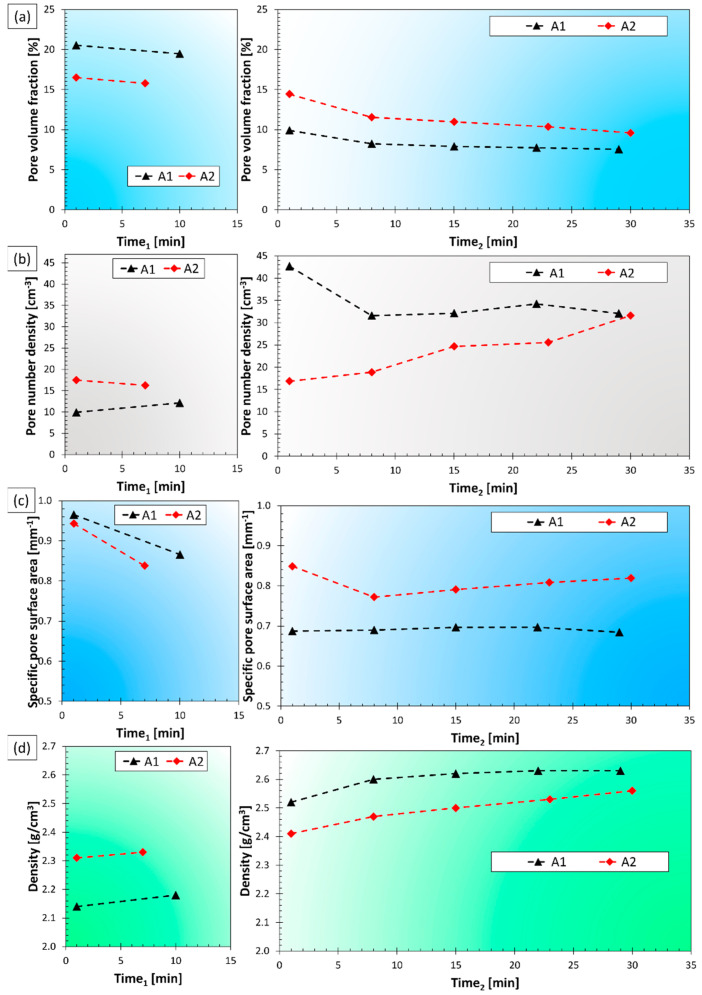
(**a**) Pore volume fraction, (**b**) pore number density, (**c**) specific pore surface area, and (**d**) density results.

**Figure 16:**
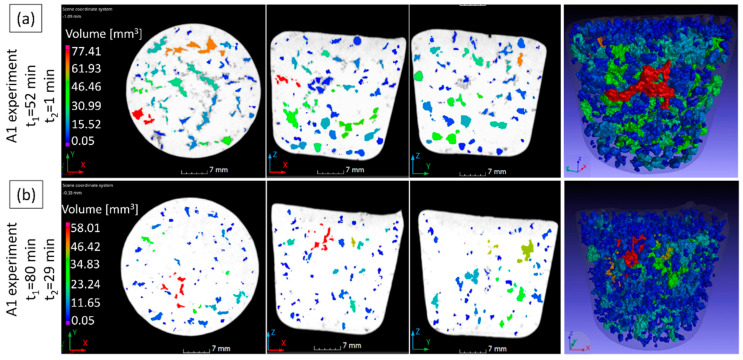
Representative CT sections and volumetric views of the pores detected in the (**a**) first and (**b**) fifth RPT sample cast after Ti-alloying during the A1 experiment.

The RPT specimens cast during the experiments were investigated with X-ray computed tomography, which is reported to be a feasible method for the characterization of RPT samples by a growing number of studies [88,89,90,91,92]. Three parameters were investigated: the pore volume fraction [%], the volumetric pore number density [cm^−3^], and the specific pore surface area [mm^−1^], which gives the surface area of pores present in 1 mm^3^ of the sample. The specific pore surface area has been named as Bifilm Spatial Index (BSI) by Song et al. [93], and according to them, this metric can be considered as an improved version of the Bifilm-Index introduced by Dispinar and Campbell [94]. The results of the CT analysis and density measurements of RPT samples are shown in Figure 15. Figure 16 shows cross-sectional and volumetric CT images of the RPT samples taken at two different time points during the A1 experiment.

During both experiments, the pore volume fraction was continuously decreased after Ti-addition, and the density was increased simultaneously (Figure 15a,d). It can be clearly seen in Figure 16, that the pore sizes were significantly reduced with time. Apparently, the Ti-addition resulted in a more significant reduction in the pore volume fraction during the A1 experiment, but it should be noted that the time difference between the time scales (time_1_ and time_2_) is not the same for the two experiments: for A1, the heating and subsequent cooling period needed an extra 24 min. From this point of view, the rate of pore volume fraction reduction was similar for both experiments. This continuous reduction of pore volume fraction can be explained by a slow natural degassing process, which involves the diffusion of H-atoms into the atmosphere above the melt. This process is highly dependent on the relative humidity of the air, the free melt surface area, the liquid metal temperature, and composition, as well as the thickness and structure of the surface oxide layer of the liquid alloy [95]. Another possibility is that the oxygen and nitrogen entrained in bifilms were gradually consumed by oxidation and consequent nitridation [96,97,98].

The pore number density results followed rather different tendencies during the two experiments (Figure 15b). In the case of the A1 experiment, the Ti-alloying at 800 °C and the subsequent cooling to 690 °C resulted in a notable increase, while during A2, the alloying step did not induce any significant change in the pore number density. Following the Ti-alloying stage, during A1, the values were first lowered and then stagnated. On the other hand, during the A2 experiment, pore number density was continuously increasing. This can be explained by the entrainment damage caused by repeated sampling. As the grain refiner particles were settled before the casting of the RPT samples began, particle sedimentation could not compensate for the effect of bifilms created during sampling.

**Figure 17:**
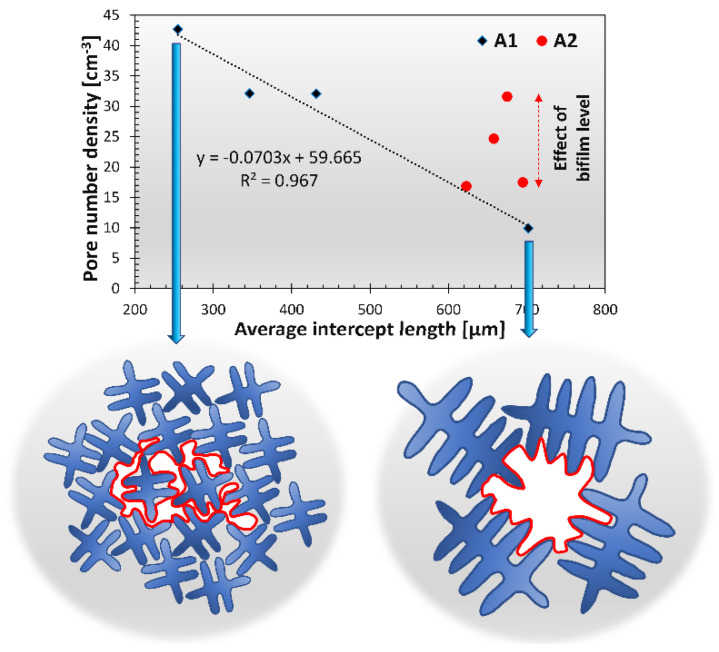
Comparison of pore number density and average intercept length (grain size) results.

The sharp increase in pore number density due to the alloying process during the A1 experiment can be explained by multiple phenomena. The manual stirring after Ti-addition, as well as the bifilm content of the master alloy, could contribute to an increased bifilm concentration, which induces a higher pore number density [99]. A more obvious explanation is the effect of grain refinement on pore number density. It is generally accepted that grain refinement results in smaller average pore sizes with an increased pore number density [71,72,100,101]. For this reason, the relationship between the grain size and the pore number density should be also investigated (Figure 17). As can be clearly seen in Figure 17, the pore number density results of experiment A1 are inversely proportional to the grain size values. For the A2 experiment, the grain size values were relatively constant, so the changes in pore number density are more clearly controlled by the bifilm quantity in the melt. 

One may ask how the effect of grain size on the pore number density can be explained if bifilm-initiated pore formation is expected to take place. This can be understood by the examination of the ability of bifilms to unfurl and inflate into pores during the solidification of the alloy. Bifilms are usually raveled into a compact form due to the bulk turbulence inside liquid alloys during melt processing [30,31,32,33]. During our experiments, the stirring applied after Ti-addition could have the same effect. Besides other important factors, such as the hydrogen content, quantity of entrained air, bifilm structure, and pressure conditions inside the semi-solid metal, pore growth is heavily affected by the solid α-Al dendrites, that can physically block the unfurling of the raveled bifilms (Figure 17). In the case of smaller grains, limited space is available for bifilms to unfurl, and only the individual folds can expand (Campbell [31] use the term “micro-inflation” for similar cases). The volume of individual pores is not necessarily different in the two cases, but when the grain size is smaller, a cluster of small pores could be found on metallographic sections and CT images, instead of the larger individual pores. For this reason, pore number density as an indicator of bifilm quantity should not be used when the α-Al grain size is changed by any kind of melt treatment.

Specific pore surface area (or Bifilm Spatial Index, BSI) results indicate a remarkable difference between the melt quality achieved during the two experiments (Figure 15c). During A1, the Ti-addition resulted in a lowered BSI value, which then stagnated, while the results of the A2 experiment show an initial decrease followed by a continuous increment, which indicates that the recurring sampling process seriously damaged melt quality.

## 4. Conclusions

This study aimed to compare the melt cleaning and grain refinement efficiency achievable by the introduction of Al_3_Ti particles into a liquid aluminum alloy. Based on the experimental results of this study, the following conclusions could be drawn:

- Effective grain refinement was realized when the blocky Al_3_Ti particles introduced by the addition of Al-10%Ti master alloy were first dissolved at 800 °C and re-precipitated at 690 °C in the form of flake-like (Al,Si)_3_Ti particles (A1 experiment).
- When the master alloy was added at a constant 690 °C (A2 experiment), the undissolved blocky Al_3_Ti particles settled within 10 min after addition. In this case, grain refinement was not achieved.
- During experiment A1, (Al,Si)_3_Ti particles heterogeneously nucleated on bifilm defects and the surface oxide layer of the melt. During their growth, particles engulfed oxide film segments. The particle nucleation on the surface oxide layer resulted in Ti-macrosegregation.
- Without dissolving the Al_3_Ti particles (A2 experiment), the melt quality was not improved by the Ti-alloying and by the increasing holding time.
- The use of pore number density of RPT samples as an indicator of bifilm quantity is inadequate when the α-Al grain size is changed.

## Figures and Tables

**Table 1:** Optical emission spectroscopy results.

	Si [wt.%]	Fe [wt.%]	Cu [wt.%]	Mn [wt.%]	Mg [wt.%]	Ti [wt.%]	Sr [wt.%]
A1/1	7.14	0.130	0.494	0.061	0.383	0.121	0.018
A1/2	7.12	0.132	0.492	0.062	0.724	0.118	0.016
A1/3	7.10	0.135	0.474	0.061	0.691	0.309	0.013
A2/1	7.13	0.128	0.473	0.059	0.379	0.130	0.017
A2/2	7.06	0.130	0.474	0.060	0.688	0.128	0.014
A2/3	7.04	0.132	0.460	0.059	0.673	0.147	0.012

**Table 2:** Sediment layer height values.

	A1	A2
The average height of the sediment layer [μm]	2251.2 ± 292.9	4881.8 ± 373.3
Minimum [µm]	1839.7	4218.1
Maximum [µm]	2944.3	5683.2

## Data Availability

Not applicable.
